# MicroRNA-1225-5p inhibits proliferation and metastasis of gastric carcinoma through repressing insulin receptor substrate-1 and activation of β-catenin signaling

**DOI:** 10.18632/oncotarget.6615

**Published:** 2015-12-14

**Authors:** Haiyin Zheng, Fuxing Zhang, Xinjian Lin, Changming Huang, Yiqin Zhang, Yun Li, Jianyin Lin, Wannan Chen, Xu Lin

**Affiliations:** ^1^ Key Laboratory of Ministry of Education for Gastrointestinal Cancer, Fujian Medical University, Fuzhou, China; ^2^ Fujian Key Laboratory of Tumor Microbiology, Fujian Medical University, Fuzhou, China; ^3^ Department of General Surgery, The First Affiliated Hospital of Xiamen University, Xiamen, China; ^4^ Department of Medicine and UC San Diego Moores Cancer Center, University of California-San Diego, La Jolla, CA, USA; ^5^ Department of General Surgery, Union Hospital of Fujian Medical University, Fuzhou, China

**Keywords:** microRNA, microRNA-1225-5p, gastric cancer, insulin receptor substrate-1, β-catenin

## Abstract

Emerging evidence has linked aberrantly expressed microRNAs (miRNAs) with oncogenesis and malignant development in various human cancers. However, their specific roles and functions in gastric carcinoma (GC) remain largely undefined. In this study we identify and report a novel miRNA, miR-1225-5p, as tumor suppressor in GC development and progression. Microarray analysis revealed that there were fifty-six differentially expressed miRNAs (thirty-two upregulated and twenty-four downregulated) in GC tumor samples compared to their corresponding nontumorous tissues. Downregulation of miR-1225-5p was frequently detected in GC and strongly correlated with more aggressive phenotypes and poor prognosis. Functional assays demonstrated that ectopic overexpression of miR-1225-5p could inhibit cell proliferation, colony formation, migration and invasion in vitro, as well as suppress tumor growth and metastasis in nude mice. Further integrative and functional studies suggested insulin receptor substrate 1 (IRS1) as a downstream effector of miR-1225-5p which acted through β-catenin signaling pathway. These results demonstrate that miR-1225-5p serves to constrain GC growth and metastatic potential via inhibition of IRS1 and β-catenin signaling. Therefore, downregulation of miR-1225-5p is likely to be one of major molecular mechanisms accounting for the development and progression of GC.

## INTRODUCTION

Gastric carcinoma (GC) is the fourth most frequent type of cancer worldwide. The highest incidence rates are in Eastern Asia, Eastern Europe, and South America, especially in China whose GC cases account for 42% of all cases in the world [1–3]. Despite therapeutic advances, the long-term survival rate for advanced GC is still low and GC remains the second most common cause of cancer death globally. Clinically, most GC patients are in the advanced stage with lymphatic, peritoneal or distal organ metastases, which misses the opportunity of radical surgery for the early stage and simultaneously predicts poor outcome [4, 5]. Early diagnosis and discovery of new therapeutic targets are crucial for a better prognosis. Although *Helicobacter pylori* infection, gastrin levels, germline mutations, dietary factors and other chronic gastric conditions are all factors involved in the development of GC, a large body of studies have shown that GC originates from a sequential accumulation of molecular and genetic alterations in stomach epithelial cells [6]. A better understanding of the molecular mechanisms for gastric tumorigenesis and progression may provide novel diagnostic and therapeutic strategies for this highly malignant tumor.

MicroRNAs (miRNAs), a class of single-strand non-coding small RNAs with 19~25 nucleotides in length, are involved in modulation of a wide array of biological processes by base-pairing, usually imperfectly, to the 3′-untranslated region(UTR) of a target messenger RNA (mRNA) leading to posttranscriptional inhibition and sometimes mRNA degradation [7, 8]. MiRNAs have been recently implicated in the regulation of tumorigenesis, differentiation, proliferation, and survival through the interference with major cellular pathways. MiRNA can play a role similar to oncogenes or tumor suppressors to regulate tumor growth, differentiation, adhesion, apoptosis, invasion and metastasis [9, 10]. Currently, link between miRNAs and gastric cancer has become increasingly apparent, and the aberrant expression of miRNAs may contribute to the initiation and progression of human GC [11, 12]. For example, miR-21, one of the major oncogenic miRNAs, is activated by *Helicobacter pylori* infection and is increased in GC [13–15]. On the contrary, the miR-29 family is downregulated in this malignancy, suggesting that it is a potential tumor suppressor of GC [16–18]. Nevertheless, further understanding of the molecular mechanisms of miRNAs in GC is needed in order to provide deeper insights into early diagnosis or better therapeutic opportunities for GC patients. In the present study, we found that fifty-six miRNAs were differentially expressed in gastric tumor samples compared with the adjacent normal gastric tissues. Among these, miR-1225-5p was demonstrated to function as a tumor suppressor since loss of miR-1225-5p expression was frequently observed in GC and strongly associated with poor patient outcome. Knockdown of miR-1225-5p promoted gastric cancer cell proliferation, invasion and xenograft tumor growth and metastasis. Mechanistically, insulin receptor substrate 1 (IRS1) was identified as a direct functional target of miR-1225-5p and consequently augmented β-catenin activity ultimately leading to the malignant phenotypes.

## RESULTS

### miRNA profiling in human gastric carcinoma

**Figure 1:**
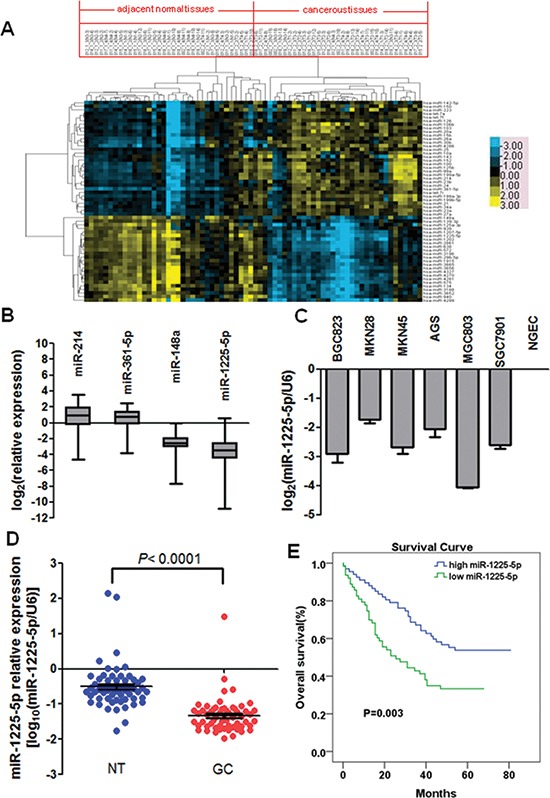
Downregulation of miR-1225-5p in gastric carcinoma (GC), which is associated with poor prognosis **A.** Unsupervised hierarchical cluster analysis of 56 differentially expressed miRNAs in 35 paired samples used for microarray analysis. The 35 paired samples are in columns and the 56 miRNAs are in rows. Blue indicates low expression and yellow indicates high expression relative to the median. T, cancerous tissue; N, adjacent normal tissue. **B**. Validation of two most differentially upregulated (miR-214 and miR-361-5p) and downregulated (miR-148a and miR-1225-5p) miRNAs in tumor and corresponding nontumorous pairs used for microarray analysis. The average miRNA expression levels in the cancerous tissues were presented in Log2 scale relative to those in the adjacent normal tissues. **C.** Downregulation of miR-1225-5p was observed in six GC cell lines. Expression levels were presented in Log2 scale compared with NGEC. NGEC, pool of nontumorous gastric mucosa tissues. **D.** miR-1225-5p was significantly downregulated in 60 primary GC compared with their corresponding nontumorous tissues. Expression was shown in log_10_ scale and was normalized against an endogenous control U6 snRNA. **E.** Downregulation of miR-1225-5p was significantly associated with poorer overall survival.

To investigate the roles of miRNA in GC, Agilent human miRNA array containing 939 human miRNA probes was performed to compare miRNA profiles between GC tumor samples and their corresponding nontumorous tissues from 35 primary GC patients. Among the 939 miRNA probes, the microarray detected 520 miRNAs in our GC pairs. With the 2-fold cut-off threshold, 56 differentially expressed miRNAs (32 upregulated and 24 downregulated) were detected in GC tumor tissues compared with their nontumorous counterparts (Table 1). Based on the above differentially expressed miRNAs, clustering was used to analyze miRNA profiles of 35 paired samples from the GC patients. As shown in Figure 1A, this unsupervised clustering successfully separated the 70 samples of cancerous tissues and adjacent normal tissues into 2 discrete groups, with the exception of 3 sample pairs (002_2_4/(002_1_4 (T4-11/N4-11), 012_2_4/(012_1_4 (T4-3/N4-3), 018_2_3/(018_1_3 (T3-11/N3-11)) that were “wrongly classified” pathologically. The microarray results were validated with quantitative PCR. The average mRNA expression levels in cancerous tissues were increased by 2.81- and 1.92-fold (p < 0.01 for both) for hsa-miR-361-5p and hsa-miR-214, but decreased by 4.76- and 6.25-fold (p < 0.01 for both) for hsa-miR-148a and hsa-miR-1225-5p relative to those in the adjacent normal tissues. The quantitative PCR results confirmed the expression of two most upregulated (hsa-miR-361-5p and hsa-miR-214) and downregulated (hsa-miR-1225-5p and hsa-miR-148a) miRNAs in the same 35 GC pairs (Figure 1B).

**Table 1:** Differential expression of miRNAs in gastric carcinomas

miRNA	Fold change
**Upregulated(n=32)**	
hsa-miR-361-5p	3.93
hsa-miR-214	3.90
hsa-miR-125b	3.59
hsa-miR-199a-3p	3.52
hsa-miR-199a-5p	3.21
hsa-miR-21	3.05
hsa-miR-100	2.91
hsa-miR-99a	2.77
hsa-miR-223	2.76
hsa-miR-103	2.74
hsa-miR-106b	2.64
hsa-miR-20a	2.63
hsa-miR-4286	2.62
hsa-miR-150	2.53
hsa-miR-199b-5p	2.53
hsa-miR-142-5p	2.47
hsa-miR-15b	2.45
hsa-let-7i	2.44
hsa-let-7f	2.44
hsa-miR-26a	2.43
hsa-miR-23a	2.34
hsa-miR-23b	2.26
hsa-miR-34a	2.24
hsa-miR-143	2.24
hsa-miR-27a	2.24
hsa-miR-24	2.20
hsa-miR-126	2.19
hsa-miR-10a	2.13
hsa-miR-152	2.10
hsa-miR-25	2.10
hsa-let-7a	2.07
hsa-miR-30b	2.07
**Downregulated(n=24)**	
hsa-miR-1225-5p	3.57
hsa-miR-1202	3.52
hsa-miR-148a	3.45
hsa-miR-572	3.11
hsa-miR-638	3.04
hsa-miR-1207-5p	3.04
hsa-miR-2861	2.92
hsa-miR-575	2.84
hsa-miR-134	2.72
hsa-miR-3656	2.61
hsa-miR-4270	2.49
hsa-miR-125a-3p	2.47
hsa-miR-4281	2.44
hsa-miR-3188	2.43
hsa-miR-4299	2.34
hsa-miR-139-3p	2.32
hsa-miR-3196	2.31
hsa-miR-939	2.25
hsa-miR-1915	2.23
hsa-miR-4327	2.21
hsa-miR-3665	2.20
hsa-miR-940	2.17
hsa-miR-3652	2.10
hsa-miR-296-5p	2.01

### miR-1225-5p is downregulated in GC and correlates with poor prognosis

Since the expression of hsa-miR-1225-5p had the largest absolute fold change from the miRNAs profiling analysis, it was chosen for all the subsequent studies. To further ascertain the downregulation of miR-1225-5p in GC, expression of miR-1225-5p in six GC cell lines and 60 pairs of primary GC was examined by quantitative PCR. Consistent with the results obtained from the miRNA microarray analysis, miR-1225-5p was markedly downregulated in both the GC cell lines and primary GC tumors (Figures 1C and 1D).

To address the clinical significance of the downregulation of miR-1225-5p in GC, the correlation of miR-1225-5p downregulation with clinicopathologic features of 130 patients was investigated. The median value of all 130 GC samples was set as the cut-off point to separate tumors with low-level expression of miR-1225-5p from those with high-level expression of miR-1225-5p. Correlation analysis showed that low-level expression of miR-1225-5p in GC was significantly associated with a more aggressive tumor phenotypes including local invasion, lymph node metastasis, advanced clinical stage and distant metastasis (Table 2). The prognostic significance of miR-1225-5p in GC was determined by Kaplan-Meier analysis demonstrating that low miR-1225-5p expression was associated with poorer overall survival in the GC cohort (Figure 1E). These results suggest that downregulation of miR-1225-5p may promote the development and progression of GC.

**Table 2:** Correlation of miR-1225-5p downregulation with clinicopathological features of 130 GC patients

Clinicopathological features	Total cases	miR-1225-5p expression		p Value
		High expression (%)	Low expression (%)	
Age(years)				
≤60	63	33(52.4)	30(47.6)	0.863
≥60	67	34(50.7)	33(49.3)	
Gender				
Male	99	52(52.5)	47(47.5)	0.837
Female	31	15(48.4)	16(51.6)	
Tumor size(cm)				
≤5	73	35(47.9)	38(52.1)	0.381
≥5	57	32(56.1)	25(43.9)	
Local invasion				
T1	21	17(81.0)	4(19.0)	0.016*
T2	23	13(56.5)	10(43.5)	
T3	33	13(39.4)	20(60.6)	
T4	53	24(45.3)	29(54.7)	
Lymph node metastasis				
N0	38	29(76.3)	9(23.7)	0.002*
N1	26	13(50.0)	13(50.0)	
N2	29	11(37.9)	18(62.1)	
N3	37	14 (37.8)	23(62.2)	
Clinical stage				
I	28	22(78.6)	6(21.4)	0.000*
II	31	20(64.5)	11(35.5)	
III	53	21(39.6)	32(60.4)	
IV	18	4(22.2)	14(77.8)	
Distant metastasis				
M0	112	63(56.3)	49(43.8)	0.01
M1	18	4(22.2)	14(77.8)	
Differentiation				
High	18	10 (55.6)	8(44.4)	0.748
Middle	64	30(46.9)	34(53.1)	
Low	33	18(54.5)	15(45.5)	
undifferentiation	15	9(60.0)	6(40.0)	

* significant difference; GC, gastric carcinoma

### miR-1225-5p suppresses GC cell growth and metastatic potential *in vitro* and *in vivo*

**Figure 2:**
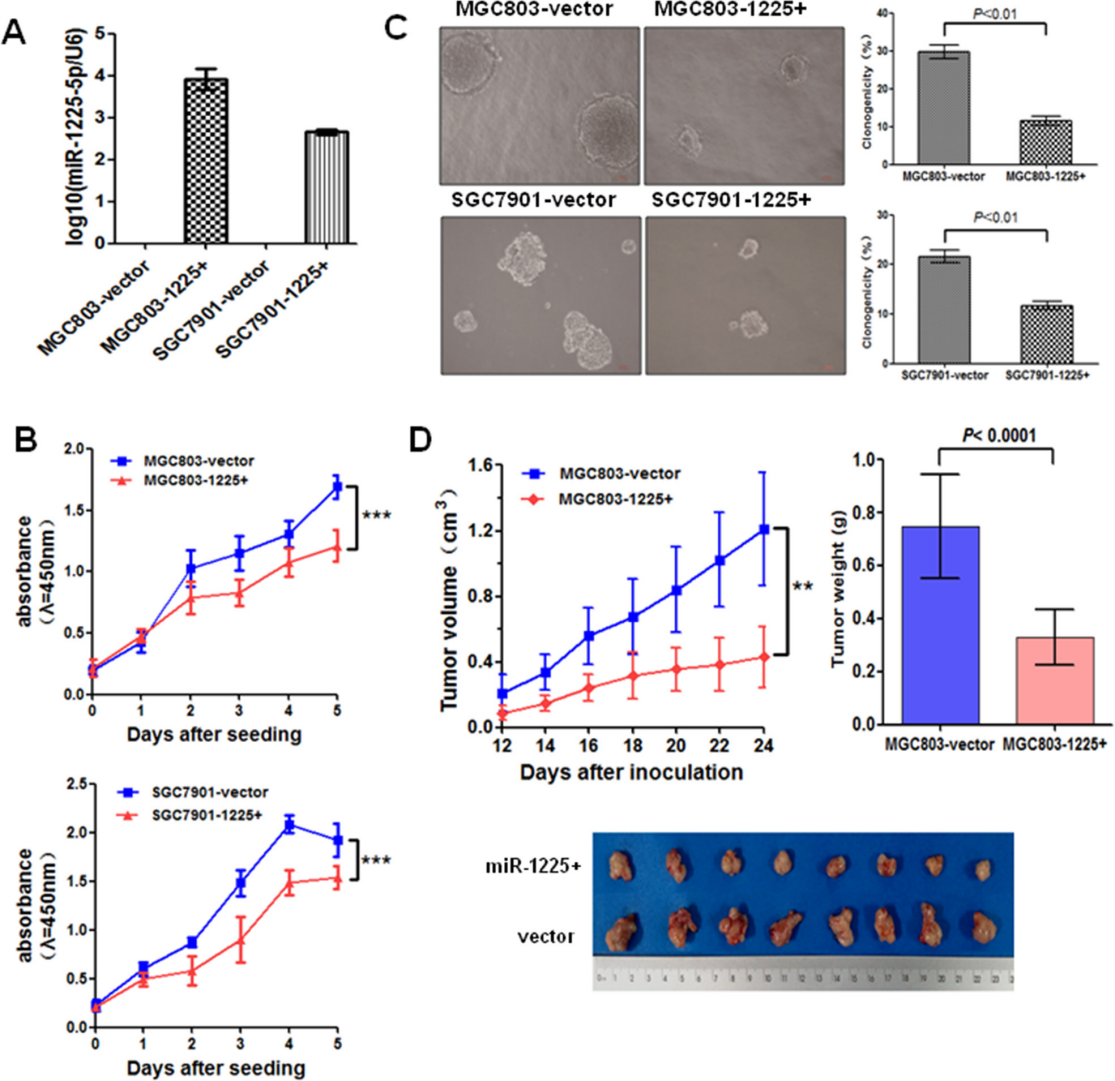
miR-1225-5p suppresses GC cell growth **A.** Relative expression of miR-1225-5p was detected by quantitative PCR in stably transfected MGC803 (MGC803-1225+) and SGC7901 (SGC7901-1225+) cells relative to empty vectors (MGC803-vector or SGC7901-vector). Expression was normalized against an endogenous control U6 snRNA. **B.** miR-1225-5p inhibited the cell growth rate in MGC803-1225+ (upper panel) and SGC7901-1225+ (lower panel) cells detected by CCK-8 assay. The results are expressed as mean ± SD of three independent experiments. ***p<0.0001. **C.** Colony formation in soft agar was significantly suppressed by miR-1225-5p. The results are expressed as mean ± SD of three independent experiments. **D.** Tumor growth curves show that miR-1225-5p inhibited xenograft tumor growth in nude mice (upper left panel). Points and bars represent the mean of eight mice with SD. Photographs of tumors removed from bodies 4 weeks after implantation (bottom panel) and the mean tumor weight of two groups measured at the end of the experiment (upper right panel). ***p<0.0001.

To explore the potential tumor-suppressive role of miR-1225-5p in GC, miR-1225-5p precursor was cloned and stably transfected into the GC cell lines MGC803 and SGC7901. The expression of mature miR-1225-5p in the stably transfected cells was confirmed by quantitative PCR (Figure 2A). As shown in Figure 2B, cell proliferation was suppressed significantly by ectopic expression of miR-1225-5p in both MGC803 and SGC7901 cells. However, enhanced expression of miR-1225-5p did not produce any change in the basal rate of cells undergoing apoptosis (Supplementary Figure S1). Colony-forming assay further confirmed the inhibitory effect of miR-1225-5p on the growth of these miR-1225-5p overexpressing cells (Figure 2C). Consistent with the results obtained from the in vitro growth assays, miR-1225-5p significantly inhibited the xenograft tumor growth in nude mice (Figure 2D). Collectively, these data indicate that miR-1225-5p acts as a novel tumor suppressor that inhibits gastric tumor growth.

**Figure 3:**
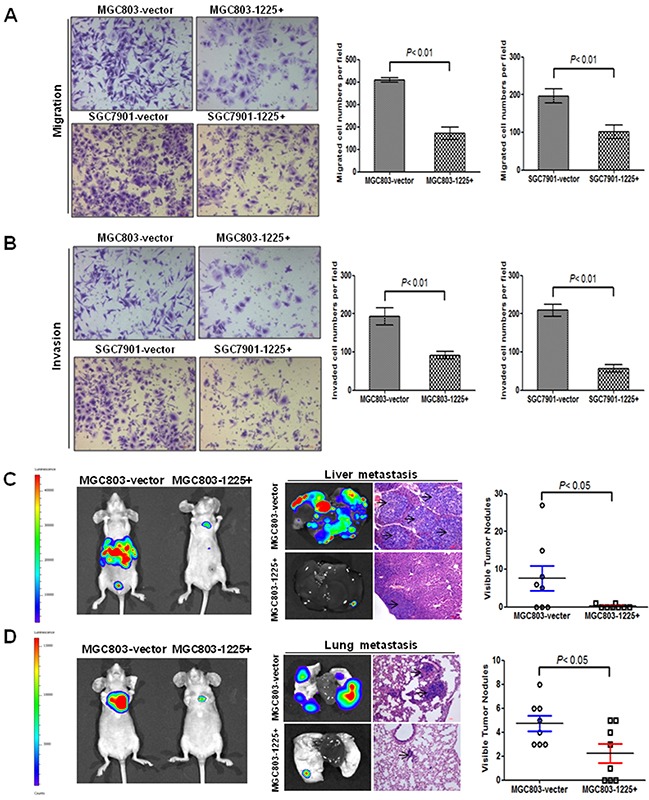
miR-1225-5p suppresses GC invasion and metastasis **A.** miR-1225-5p inhibited migration of MGC803-1225+ (upper right panel) and SGC7901-1225+ (lower right panel) cells (p<0.001). The results are expressed as mean ± SD of three independent experiments. **B.** miR-1225-5p inhibited invasion of MGC803-1225+ (upper right panel) and SGC7901-1225+ (lower right panel) cells (p<0.001). The results are expressed as mean ± SD of three independent experiments. **C.** miR-1225-5p suppressed GC cell metastasis in vivo. Representative bioluminescence images of whole mouse and the livers of the BALB/c nude mice 42 days after tail vein injection of 2 × 10^6^ MGC803-1225+ or MGC803-vector cells, showing less metastasis when miR-1225-5p was expressed. **D.** Representative bioluminescence images of whole mouse and mouse lung lobes showing that expression of miR-1225-5p decreased the extent of metastasis to lung when imaged at 42 days after the tail vein injection.

The effect of miR-1225-5p on cell migration and invasive potential was examined using the Boyden Transwell chamber assays. Overexpression of miR-1225-5p in MGC803-1225+ and SGC7901-1225+ cells substantially impaired the cell migratory ability as compared with the empty vector-transfected cells (Figure 3A). Similarly, miR-1225-5p significantly decreased GC cell invasion through the Matrigel-coated transwell inserts (Figure 3B). The impact of miR-1225-5p on metastatic potential in vivo was assessed by molecularly engineering the MGC803-1225+ cells to express the luciferase protein and then injecting 2 × 10^6^ cells into the tail vain of BALB/c nude mice. Bioluminescence imaging was used to serially monitor the appearance of metastases in living mice. After 6 weeks, the mice were sacrificed and metastatic nodules were counted on the surface of liver. The number of nodules formed in the liver of the mice injected with MGC803-1225+ cells was remarkably less compared to the mice injected with the control MGC803-vector cells (Figure 3C). Notably, seven out of ten mice in MGC803-1225+ group did not form any metastatic lesions. Although no visible metastatic nodules were observed on the surface of the lungs, the presence of micrometastases in the lungs can be detected by haematoxylin and eosin staining that showed significant reduction of the lung metastases in the mice injected with MGC803-1225+ cells (Figure 3D). These results clearly indicate that miR-1225-5p functions to limit migration, invasion and metastasis as well.

### IRS1 is a direct target of miR-1225-5p

**Figure 4:**
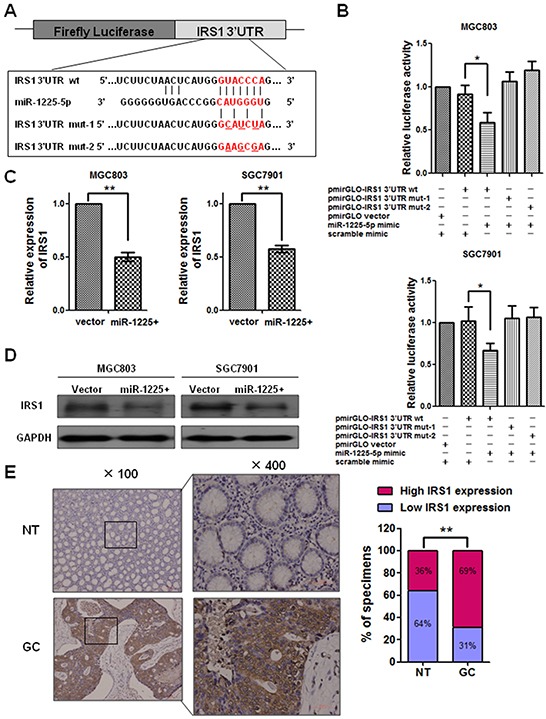
IRS1 is a direct target of miR-1225-5p **A.** The predicted binding sequence of human hsa-miR-1225-5p and its binding site in the 3′-untranslated region (UTR) of IRS1 (wt) was presented for alignment. Mut-1 and mut-2 were two mutant sequences of 3′-UTR of IRS1 with the binding sites of miR-1225-5p. **B.** Luciferase assay was used to confirm the interaction of miR-1225-5p with IRS1. 3′-UTR of IRS1 containing the target binding site (wt) and two mutant sequences of 3′-UTR of IRS1 (mut-1 and mut-2) were cloned into downstream of a firefly luciferase gene. The plasmids (wt or mut) were co-transfected with miR-1225-5p mimic or scramble mimic. PmirGLO vector was co-transfected with miR-1225-5p mimic and scramble mimic as positive control. Expression was normalized against Renilla luciferase activity. +, plasmid or mimic added; -, no plasmid or mimic added. *p<0.05. **C.** Quantitative PCR result shows that IRS1 was downregulated by miR-1225-5p in MGC803-1225+ (left panel) and SGC7901-1225+ (right panel) cells (**p<0.01). Expression was normalized against an endogenous control GAPDH and results are expressed as mean ± SD of three independent experiments. **D.** Western blot analysis shows that miR-1225-5p inhibited the protein expression of IRS1 in MGC803-1225+ (left panel) and SGC7901-1225+ (right panel) cells. GAPDH was used as an internal control. **E.** Immunohistochemical staining result shows the expression of IRS1 was upregulated in GC tissues compared with nontumorous tissues. Two representative cases are shown. Percentage of specimens shows low or high IRS1 expression in tumor and corresponding nontumorous tissues. **p<0.01.

To understand the mechanisms underlying miR-1225-5p induced inhibition of GC growth and metastasis, the bioinformatics algorithms of TargetScan, pictar and miRanda were employed to predict potential targets by compiling all the predicted genes for functional clustering analysis classified by Gene Ontology and KeGG pathway database. Among the predicted targets, ETV1, IRS1, FUS and CSK were found to be associated with tumorigenesis and metastasis. To investigate whether the four genes could be regulated by miR-1225-5p, we constructed the vector containing the 3′-UTR of each gene downstream of the firefly luciferase reporter and cotransfected with miR-1225-5p mimic into MGC803 and SGC7901 cells. Interestingly, luciferase activity was significantly reduced in those cells cotransfected with IRS1 3′-UTR sequence and miR-1225-5p but not in the cells with other three genes 3′-UTR sequence and miR-1225-5p (Supplementary Figure S2). More importantly, an interaction between miR-1225-5p and IRS1 3′-UTR was specific as introduction of mutated IRS1 3′-UTR together with miR-1225-5p mimic into MGC803 cells did not alter the luciferase activity (Figure 4A, 4B). Then, quantitative PCR and western blot analysis were conducted to measure the expression level of IRS1. The result showed that the expression of IRS1 mRNA and protein was downregulated in miR-1225-5p treated cells (Figure 4C, 4D). Furthermore, immunohistochemical staining was performed to measure the levels of IRS1 protein in GC specimens and matched normal gastric tissues. Figure 4E showed that the high-level IRS1 expression was present in 69% GC tumor tissues versus 36% of corresponding non-tumor tissues (p < 0.01). These results implicate that miR-1225-5p might directly target IRS1 and expression of IRS1 is correlated with gastric tumorigenesis.

### Enhanced expression of IRS1 reverses the effect of miR-1225-5p

**Figure 5:**
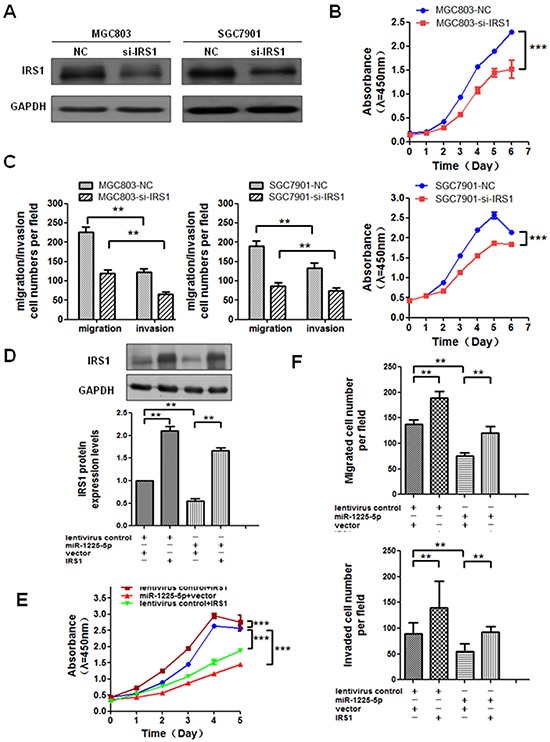
Enhanced expression of IRS1 reverses the effect of miR-1225-5p **A.** A specific small interfering RNA against IRS1 was introduced into MGC803 and SGC7901 cells. Inhibition was confirmed by western blot analysis in MGC803 (left panel) and SGC7901 (right panel) cells. GAPDH was used as an internal control. **B.** Cell growth rate was inhibited when IRS1 was knocked down in GC cells. The results are expressed as mean ± SD of three independent experiments. ***p<0.0001. **C.** Migratory and invasive capabilities of GC cells were both suppressed after knockdown of IRS1. The results are expressed as mean ± SD of three independent experiments. **p<0.01. **D.** MGC803-vector and MGC803-1225+ cells were respectively transfected with IRS1-expressing plasmid or empty plasmid. Western blot shows MGC803-1225+ cell transfected with IRS1-expressing plasmid blocked miR-1225-5p-induced suppression of IRS1 protein expression. GAPDH served as an internal control. lentivirus control, MGC803-vector cell; miR-1225-5p, MGC803-1225+ cell; vector, pcDNA3.1/myc-His(−)A empty plasmid; IRS1, IRS1-expressing plasmid; +, plasmid added; —, no plasmid added. The results are expressed as mean ± SD of three independent experiments. *p<0.05. **E.** Cell growth curves by CCK-8 assay show that IRS1 restoration abrogated miR-1225-5p-induced suppression of proliferation in MGC803-1225+ cell after transfected with IRS1-expressing plasmid. The results are expressed as mean ± SD of three independent experiments. ***p<0.0001. **F.** Migration and invasion assays show that IRS1 restoration abolished miR-1225-5p-induced suppression of invasion and metastasis in MGC803-1225+ cell after transfected with IRS1-expressing plasmid. The results are expressed as mean ± SD of three independent experiments. **p<0.01.

Given that IRS1 may be a direct target of miR-1225-5p and possibly involve in gastric tumorigenesis, we first asked whether downregulation of IRS1 could suppress GC cell growth and reduce their invasive potential. Expectedly, siRNA-mediated knockdown of IRS1 in MGC803 or SGC7901 cells, as confirmed by Western blot analysis (Figure 5A), significantly inhibited the cell growth, migration and invasion (Figures 5B and 5C). To investigate the relationship of IRS1 and miR-1225-5p in regulation of cell proliferation, migration and invasion, IRS1-expressing plasmid was introduced into MGC803-1225+ cells (Figure 5D) for assessment of phenotypic changes. The enhanced expression of IRS1 significantly increased or reversed the proliferative, migratory and invasive capabilities of MGC803-1225+ cells (Figures 5E and 5F). Taken together, these results suggest that the tumor-suppressive effect of miR-1225-5p is mediated, at least in part, through inhibition of IRS1.

### IRS1 regulates β-catenin expression and its target gene expression

**Figure 6:**
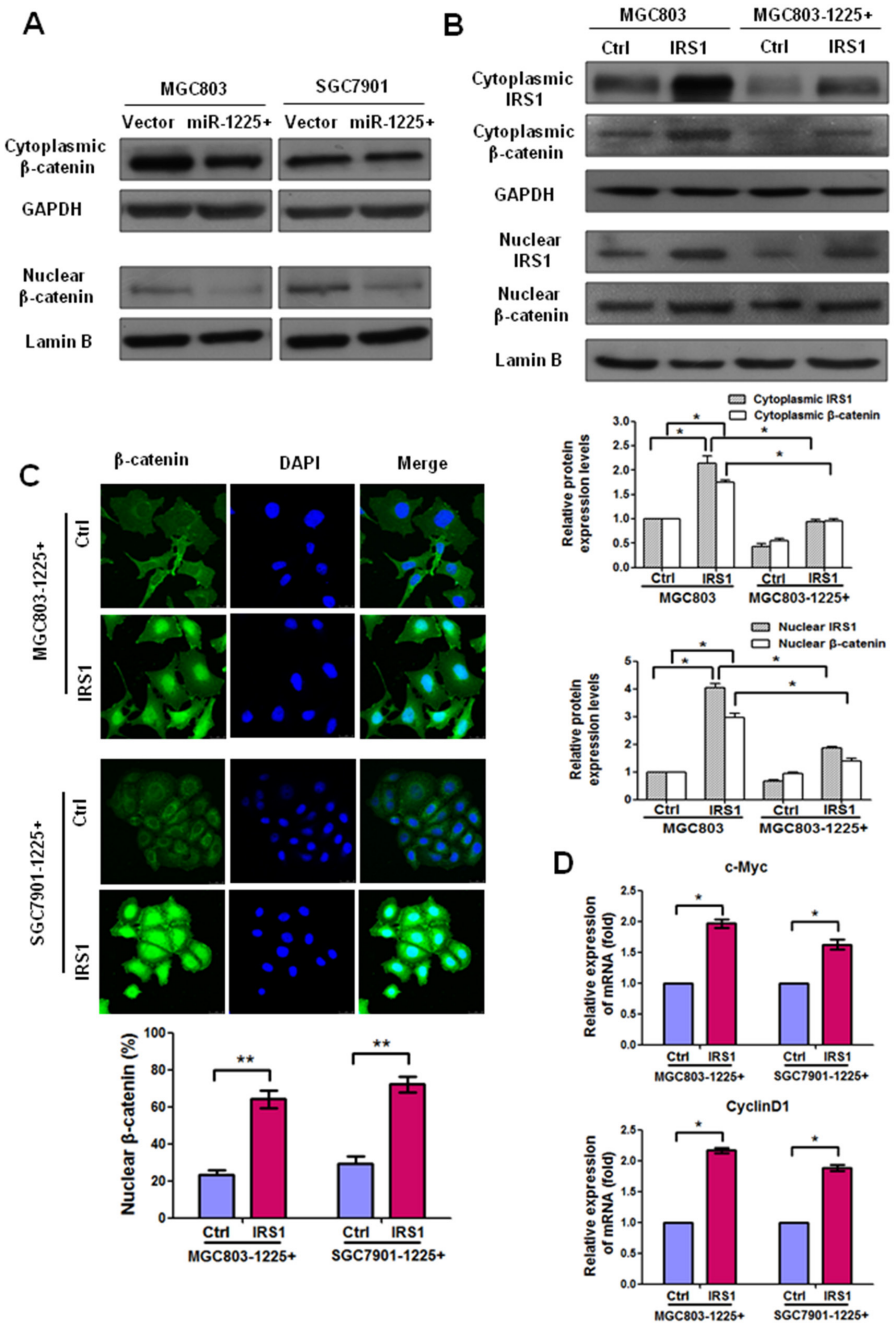
IRS1 increases translation of β-catenin and its target gene expression **A.** Western blot analysis shows that miR-1225-5p decreased the protein levels of nuclear and cytoplasmic β-catenin in MGC803-1225+ (left panel) and SGC7901-1225+ (right panel) cells. **B.** Western blot analysis shows that ectopic expression of IRS1 enhanced the expression of nuclear and cytoplasmic β-catenin expression after IRS1-expressing plasmid was introduced into MGC803 or MGC803-1225+ cells as compared with control cells. Nuclear and cytoplasmic protein loading was normalized for equal levels of Lamin B and GAPDH, respectively. The results are expressed as mean ± SD of three independent experiments. *p<0.05. **C.** Immunofluorescence staining showed that β-catenin was predominantly localized in the nucleus in IRS1-expressing MGC803-1225+ or SGC7901-1225+ cells than control cells. Expression of β-catenin is shown in green. 4′,6′ Diamino-2-phenylindole (DAPI) (blue) was used as a nuclear counterstain. Original magnification, ×400. The percentage of nuclear β-catenin-positive staining cells in at least total 200 cells is presented as mean ± SD. **p<0.01. **D.** qPCR results indicated mRNA upregulation of β-catenin downstream target genes (c-Myc and cyclin D1) in MGC803-1225+ cells or SGC7901-1225+ cells transfected with IRS1-expressing plasmid as compared with control plasmid. *p<0.05. The bar graphs represent the mean ± SD of three independent experiments.

IRS1 has been shown to promote Wnt-β-catenin signaling which induce transcription of downstream genes related to tumor growth and metastasis [19]. Western blot analysis showed that the expression of both cytoplasmic and nuclear β-catenin in MGC803-1225+ and SGC7901-1225+ cells was significantly reduced as compared with empty vector-transfected cells (Figure 6A), indicating that there was an association between the expression levels of miR-1225-5p and activation of β-catenin in GC cells.

Our next step was to investigate if miR-1225-5p inhibits β-catenin through suppression of IRS1. Therefore, we transiently expressed IRS1 in MGC803 or MGC803-1225+ cells. As expected, western blot analysis of nuclear and cytoplasmic protein fractions from MGC803 or MGC803-1225+ cells transfected with control or IRS1-expressing plasmid indicated more nuclear and cytoplasmic β-catenin expression in IRS1-expressing cells than control cells (Figure 6B). Consistent with the western blot results, the immunofluorescence data showed that the expression levels of both cytoplasmic and nuclear β-catenin increased in IRS1-expressing MGC803-1225+ or SGC7901-1225+ cells than control cells (Figure 6C). In addition, considering that overexpression of miR-1225 decreased both mRNA and protein level of β-catenin (data not shown) together with the finding that miR-1225-5p could decrease the expression of IRS1, one may speculate that miR-1225-5p was able to transcriptionally downregulate β-catenin through decreasing the expression of IRS1. This observation was in accordance with a previous report showing that β-catenin could be transcriptionally regulated by IRS1/2 [20]. Collectively, these data indicate that downregulation of miR-1225-5p in GC is associated with activation of β-catenin through upregulation of IRS1.

To investigate whether the IRS1-induced increase translation of β-catenin leads to upregulation of its downstream targets such as c-Myc and cyclin D1, we evaluated mRNA expression of these targets in MGC803-1225+ or SGC7901-1225+ cells. Quantitative PCR results revealed a significant increase in the mRNA levels of *Ccnd1* and *c-Myc* in MGC803-1225+ cells or SGC7901-1225+ cells transfected with IRS1-expressing plasmid as compared with control plasmid (Figure 6D). Taken together, these data demonstrated that IRS1 functions as a downstream effector of miR-1225-5p and positively regulates β-catenin activity and downstream targets.

## DISCUSSION

An increasing number of studies have shown that aberrant expression of miRNAs is correlated with the development and progression of various human cancers. In order to elucidate the role and function of deregulated miRNAs in cancer, several methods including microarray and qRT-PCR techniques have been used in such investigations [21–23]. In the present study, human miRNA array was employed to investigate differentially expressed miRNAs between GC tumors and their corresponding nontumorous tissues from thirty-five primary GC patients. Fifty-six differentially expressed miRNAs were detected. Two most upregulated (hsa-miR-361-5p and hsa-miR-214) and two most downregulated (hsa-miR-1225-5p and hsa-miR-148a) miRNAs were further validated using qRT-PCR in GC tissue pairs. Among them, previous reports have established the association of hsa-miR-148a, 214 and 361-5p with human cancers. For example, miR-148a has been shown to serve as a tumor suppressor in cancers of the liver [24, 25], prostate [26, 27], colon [28, 29], stomach [30], pancreas [31, 32], bladder [33], ovary [34] and breast [35, 36]. MiR-148a was significantly downregulated in both GC cell lines and GC tissue samples compared to the adjacent normal gastric tissues [37–39] which is consistent with our findings in this study. The role of miR-214 in human cancers appears contradictory. Upregulation of miR-214 is present in pancreatic, hepatoblastoma, gastric, osteosarcoma, esophageal, ovarian, bladder and melanoma cancer whereas its downregulation could be found in some other caner types including hepatocellular, cervical, breast, prostate cancer [40]. These suggest that miR-214 may function as either a tumor suppressor or tumorigenic miRNA. MiR-361-5p was also often aberrantly expressed in some human cancers, such as cervical and prostate cancer [41, 42]. Consistently, in this study both miRNA array and quantitative PCR analysis showed that there was a significant increase in the level of miR-361-5p expression between GC tumors and their corresponding nontumorous tissues. We found that miR-1225-5p was downregulated in primary GC tumors with a highest absolute fold change. MiR-1225-5p is a newly identified and rarely reported miRNA. Exploration of its function and pathogenicity would not only aid in deeper understanding of the pathogenesis of GC, but also provide an opportunity for discovery of a novel molecular biomarker and therapeutic target for the diagnosis and treatment of GC.

In the present study, we demonstrated that miR-1225-5p was significantly downregulated in six GC cell lines and GC tissue samples as compared to the normal gastric tissues. We further analyzed the correlation of miRNAs expression with clinicopathologic characteristics. A robust association between low miR-1225-5p expression in tumors with advanced clinical stage, depth of local invasion, lymph node metastasis, and distant metastasis was confirmed in 130 patients with GC. These findings suggest that miR-1225-5p plays a crucial role in initiation and development of GC. Moreover, we showed that low miR-1225-5p expression in GC tumors was associated with poorer overall survival. Altogether, we assume that miR-1225-5p function as a tumor suppressor in GC and may be exploited as a prognostic biomarker in GC.

To address the issue of whether miR-1225-5p has an effect on the development and progression of GC, we re-expressed miR-1225-5p in GC cell lines MGC803 and SGC7901, both of which have a low basal level of miR-1225-5p expression. Re-expression of miR-1225-5p effectively slowed tumor cell growth, and inhibited colony formation as well as suppressed GC xenograft tumor growth in nude mice. In addition, re-expression of miR-1225-5p decreased tumor cell motility and invasion. Consistently, a metastasis experimental animal model also confirmed that miR-1225-5p significantly reduced the colonization of metastatic tumors in the liver and lungs. These data points toward an important role of miR-1225-5p, similar to tumor suppressor, in GC cell growth, invasion and metastasis,

MiRNAs may exert diverse biological functions by regulating the expression of downstream target genes in different tissues or cancers. MiRNAs can negatively regulate many target genes through binding their 3′-UTR in order to induce cleavage of the message or inhibit translation. Hence, one important task is to identify the downstream target genes regulated by the dysregulated miRNA. In our study, prediction of the candidate target genes for miR-1225-5p was performed by bioinformatics tools (Targetscan, PicTar and MiRanda). Then, IRS1, one of putative target genes, was verified by luciferase reporter assay, quantitative PCR and western blot analysis. We found that miR-1225-5p could directly bind the 3′-UTR of IRS1 and over-expression of miR-1225-5p downregulated IRS1 expression in both mRNA and protein level. Furthermore, enhanced expression of IRS1 promoted GC cell growth and invasion, and more importantly, blocked miR-1225-5p-induced suppression of cell proliferation and invasion. These results suggest a strong negative regulation between miR-1225-5p and IRS1 in GC.

IRS1, the first identified member of IRS family, is frequently overexpressed in many solid tumors such as hepatocellular, prostate, colorectal and mammary cancer [43–46]. IRS1 serves as a signal adaptor protein in oncogenic transformation by coordinating and amplifying numerous signals within the tumor cells. IRS1 has also been shown to interact with β-catenin whose activation induces the expression of several genes involved in tumor growth and metastasis [19]. In this study, we found that expression of β-catenin in miR-1225-5p overexpressed GC cells was significantly reduced. Furthermore, restoration of IRS1 expression in MGC803-1225+ or SGC7901-1225+ cells enhanced β-catenin translation and induced an increase of mRNA and protein expression of β-catenin target genes, c-Myc and cyclin D1. Results obtained from these studies clearly indicate that downregulation of miR-1225-5p in GC is associated with upregulation of IRS1 and activation of β-catenin signaling pathway, thus contributing to the tumor growth and metastasis.

In summary, this is the first study to explore the role of miR-1225-5p in cancer and demonstrate that miR-1225-5p is likely to function as a tumor suppressor specifically in GC. Downregulation of miR-1225-5p in GC tumors, which is strongly correlated with tumor progression and clinical prognosis, merits further development as a diagnostic and prognostic biomarker. In addition, miR-1225-5p may be a potential target in cancer therapy since it regulates IRS1 and consequent β-catenin pathway, a frequent and crucial signaling event associated with malignant transformation.

## MATERIALS AND METHODS

### Cell lines and clinical samples

Six human GC cell lines (BGC823, MKN28, MKN45, AGS, MGC803, SGC7901) were obtained from the Type Culture Collection of the Chinese Academy of Sciences (Shanghai, China). All cell lines were maintained in DMEM supplemented with 10% FBS except AGS in Ham's F12 medium. Human GC samples and their corresponding adjacent nontumorous gastric tissues were collected at the time of surgical resection at the First Affiliated Hospital and Union Hospital of Fujian Medical University (Fuzhou, China) from 2008 to 2009. All samples were immediately frozen and stored in liquid nitrogen or fixed in 10% formalin for paraffin embedding. All samples were collected with patients’ informed consent and the study was approved by the institutional review board and regulatory authorities of Fujian Medical University.

### miRNA microarray analysis

MiRNAs were extracted from 35 pairs of GC tumor and corresponding nontumorous samples and hybridized on miRNA microarray chip (Agilent Technologies, Santa Clara, California, USA) containing 939 human miRNA probes found in the miRNA Registry (http://www.mirbase.org/; accessed miRBase R16.0, Sept 2010). Clinical information about the samples is presented in Supplementary Table S1. Microarray chip analysis was performed by CapitalBio (CapitalBio Corp, Beijing, China). Procedures were performed as described in detail on the website of CapitalBio (http://www.capitalbio.com). Gene expression data were normalized and analyzed in GeneSping software (Agilent Technologies, USA). The fold change was determined by calculating the expression of miRNA in GC tumors and corresponding nontumorous tissues in a log_2_ format. Clustering analysis was performed by Cluster 3.0 software (Stanford University, California, USA).

### RNA extraction and real-time quantitative PCR

Total RNA was extracted from cultured cells or frozen tissues using the miRNeasy Mini Kit (Qiagen, Hilden, German) and 1 mg RNA was reverse transcribed using miScript Reverse Transcription Kit (Qiagen, Hilden, German) for first strand complementary DNA synthesis. Quantitative PCR was then carried out using a miRNA-specific miScript Primer Assay (Qiagen) in combination with the miScript SYBR Green PCR Kit (Qiagen) with the StepOne™ realtime PCR System (Applied Biosystems, Carlsbad, California, USA). MiRNAs (Hsa-miR-361-5p, hsa-miR-214, hsa-miR-1225-5p and hsa-miR-148a miR-1225-5p) are amplified using the miScript Universal Primer (provided in the miScript SYBR Green PCR Kit), which primes from the universal tag sequence, together with the miScript Primer Assay specific for the mature miRNA under study. SNORD6 (U6 snRNA) was used as an internal control. Quantitative PCR of mRNA was performed using SYBR Premix EX Taq kit (Takara, Shiga, Japan). The expression level was normalized against endogenous GAPDH.

### Plasmids and generation of stably transduced cell lines

Construction of the miR-1225-5p expression plasmid and packaging lentivirus were provided by Genechem Biotechnology (Shanghai, China). Sigma, St Louis, Missouri, USA). After infection, puromycin at 1.5 μg/ml (Sigma) was used to select stably transduced cells over a 10-day period. Stable miR-1225-5p overexpressing clones (MGC803-1225+ or SGC7901-1225+) and empty vector-transfected cells (MGC803-vector or SGC7901-vector) with a low basal level of miR-1225-5p expression were selected for further study. The expression of miR-1225-5p in the stable cell lines was confirmed by quantitative PCR.

The open reading frames (ORFs) of IRS1 were PCR amplified and cloned into mammalian expression vector pcDNA.3.1/myc-His(−)A (Invitrogen) to generate IRS1 expression vectors. The wild-type and mutated 3′-UTRs of the predicted targets ETV1, IRS1, FUS and CSK were amplified and cloned downstream to the luciferase gene in a pmirGLO Dual-Luciferase miRNA Target Expression Vector (Promega, Madison, USA), respectively. Recombinant plasmid transfection was performed with Roche, Basel, Switzerland). The primers used are shown in Supplementary Table S2.

### Oligonucleotide transfection

MiR-1225-5p mimic was purchased from Dharmacon (Lafayette, Colorado, USA). SiRNA Duplex Oligonucleotides Mix targeting human IRS1 mRNA was purchased from Santa Cruz (California, USA). Oligonucleotide transfection was performed with X-tremeGENE siRNA reagent (Roche).

### Cell proliferation assay

Cell proliferation was assessed using the Cell Counting Kit-8 (CCK-8; Dojindo, Kuma-moto, Japan). The stable miR-1225-5p overexpressing (MGC803-1225+ and SGC7901-1225+) or empty vector-transfected cells were seeded at a density of 5× 10^3^ cells per well in 96-well plates and incubated at 37°C, 5% CO_2_ for 24, 48, 72 or 96 h. 10 mL of CCK-8 solution was added into each well and incubated at 37°C for 4 h. The absorbance at 450 nm was measured using a microplate reader.

### Soft agar colony formation assay

2 × DMEM containing 20% FBS and 5 × 10^3^ cells was mixed with equal volume of 0.7% agarose and immediately plated in 6-well plates containing an underlayer of 0.5% agarose made in 1× DMEM supplemented with 10% FBS. The plates were cultured at 37°C under 5% CO_2_ for 5 to 21 days. Surviving colonies (>50 cells per colony) were counted and photographed with a Qimaging Micropublisher 5.0 RTV microscope camera (Olympus, Tokyo, Japan).

### Cell migration and invasion assay

For the migration assay, 5 × 10^4^ cells in serum-free media were placed into the upper chamber of an insert (8-mm pore size; BD Bioscience). For the invasion assay, the transwell insert was coated with Matrigel (BD Bioscience) and 8 × 10^5^ cells were plated onto the top of the coated filters. The medium containing 20% fetal bovine serum was placed to the lower chamber to act as a chemoattractant. After several hours of incubation, the cells that did not migrate or invade through the pores of the transwell insert were removed with cotton swabs and then the insert was stained with 0.1% crystal violet in 20% methanol, imaged, and counted using an a Qimaging Micropublisher 5.0 RTV microscope camera (Olympus).

### Animal studies

For in vivo tumor growth study, 2 × 10^6^ miR-1225-5p overexpressing MGC803-1225+ cells and the control MGC803-vector cells were inoculated subcutaneously on the left and right flank region, respectively, of 4~6-week-old BALB/c nude mice (eight mice per group). Tumor volume was measured every 2 days and calculated by the formula: length × width^2^/2 and plotted as a function of time to generate the in vivo growth curves. At an endpoint, tumors were excised and embedded in paraffin for haematoxylin and eosin staining.

For in vivo metastasis study, 2 × 10^6^ MGC803-1225+ and MGC803-vector cells engineered to express luciferease were injected intravenously into the tail vein of 4~6-week-old BALB/c nude mice (ten mice per group). Bioluminescence imaging was performed using the Xenogen IVIS Spectrum Imaging System (Caliper Life Sciences) to detect the formation of metastatic tumors in living animals. Fifteen minutes prior to imaging, mice were i.p. injected with 150 mg/kg luciferin (Promega). Following anesthesia, images were taken and analyzed using Spectrum Living Image 4.0 Software (Caliper Life Sciences). After 6 weeks, the number of tumor nodules formed on the liver surfaces was counted. Lungs and livers were excised and embedded in paraffin for further histopathological analysis. All animal studies were approved by the Fujian Medical University Institutional Animal Care and Use Committee.

### Dual-luciferase reporter assay

GC cells (MGC803 or SGC7901) were seeded into 24-well plates. After 18~24 h incubation, 1 mg pmirGLO report vector carrying wild type 3′-UTR or mutated 3′-UTR of miR-1225-5p targets was cotransfected with 100 pmol negative control or miR-1225-5p mimic into the GC cells using X-tremeGENE reagent. Forty-eight hours after transfection, firefly and Renilla luciferase activities were measured with a Dual-Luciferase Reporter System (Promega). PmirGLO report vector was used as a positive control.

### Immunofluorescence assay

For immunofluorescence staining, cells were fixed in 4% ice-cold paraformaldehyde and permeabilized using 0.5% Triton X-100/PBS for 20 min at room temperature. The cells were blocked with 10% normal goat serum (ZSGB Biotech, China) for 1 h. β-catenin was stained with anti-β-catenin rabbit monoclonal antibody (Abcam, 1:50 dilution) overnight at 4°C. Cells were then rinsed with PBS and incubated with Alexa Fluor 488 conjugated goat anti-rabbit secondary antibody (1:200, 2 mg/mL, Invitrogen, Carlsbad, CA, USA). DAPI (2 mg/mL, Invitrogen) was used to stain nuclei and cells were examined by a laser scanning confocal microscope (Leica, Germany).

### Immunohistochemistry

Immunohistochemical staining was carried out following standard streptavidin-biotin-peroxidase complex method. Briefly, tissue sections were deparaffinized, and nonspecific bindings were blocked with 10% normal goat serum for 30 minutes. The section was then incubated with anti-IRS1 rabbit monoclonal antibody (Abcam, 1:200 dilution) at 4°C overnight. Slides were then incubated with biotinylated goat anti-rabbit secondary antibody at room temperature for 15 minutes. Finally, the slides were incubated with DAB for 5 min and then counterstained with hematoxylin for 1 minute. The degree of immunostaining of indicated proteins was evaluated and scored by 2 independent observers. Scores representing the proportion of positively stained tumor cells were graded as: 0 (no positive tumor cells); 1 (<10%); 2 (10%–50%); and 3 (>50%). The intensity of staining was determined as: 0 (no staining); 1 (weak staining = light yellow); 2 (moderate staining = yellow brown); and 3 (strong staining = brown). The staining index (SI) was calculated as the product of staining intensity × percentage of positive tumor cells, resulting in scores of 0, 1, 2, 3, 4, 6, and 9. The cutoff was identified as an SI score greater than or equal to 4, which was considered to be high expression, and less than or equal to 3, which was considered to be low expression.

### Western blot analysis

Western blotting was performed according to the standard protocol with antibodies against IRS1 (Abcam, 1:1000 dilution) and β-catenin (Abcam, 1:1000 dilution). GAPDH (Sigma, 1:5000 dilution) was used as an internal control. Nuclear extracts were prepared using the Nuclear Extraction Kit (Sangon Biotechology, Shanghai, China) according to the manufacturer's instructions. Lamin B (Santa Cruz Biotechnology, 1:200 dilution) served as a nuclear internal control.

### Statistical analysis

Statistical analysis was carried out using SPSS 21.0 for Windows. Student's t test was used to analyze the results expressed as mean ± SD. The χ^2^ test or Fisher's exact test was used to analyze the association of miR-1225-5p expression and clinical-pathological parameters. The survival curves were plotted using Kaplan-Meier analysis. Differences were considered significant when the p value was less than 0.05.

## SUPPLEMENTARY FIGURES AND TABLES



## References

[1] Ferlay J, Shin HR, Bray F, Forman D, Mathers C, Parkin DM (2010). Estimates of worldwide burden of cancer in 2008: GLOBOCAN 2008. Int J Cancer.

[2] Yang L (2006). Incidence and mortality of gastric cancer in China. World journal of gastroenterology.

[3] Leung WK, Wu MS, Kakugawa Y, Kim JJ, Yeoh KG, Goh KL, Wu KC, Wu DC, Sollano J, Kachintorn U, Gotoda T, Lin JT, You WC (2008). Screening for gastric cancer in Asia: current evidence and practice. Lancet Oncol.

[4] Orditura M, Galizia G, Sforza V, Gambardella V, Fabozzi A, Laterza MM, Andreozzi F, Ventriglia J, Savastano B, Mabilia A, Lieto E, Ciardiello F, De Vita F (2014). Treatment of gastric cancer. World journal of gastroenterology.

[5] Roukos DH (2000). Current status and future perspectives in gastric cancer management. Cancer Treat Rev.

[6] Yasui W, Sentani K, Sakamoto N, Anami K, Naito Y, Oue N (2011). Molecular pathology of gastric cancer: research and practice. Pathol Res Pract.

[7] Ambros V (2004). The functions of animal microRNAs. Nature.

[8] Lewis BP, Shih IH, Jones-Rhoades MW, Bartel DP, Burge CB (2003). Prediction of mammalian microRNA targets. Cell.

[9] Hata A, Lieberman J (2015). Dysregulation of microRNA biogenesis and gene silencing in cancer. Sci Signal.

[10] Esquela-Kerscher A, Slack FJ (2006). Oncomirs - microRNAs with a role in cancer. Nature reviews Cancer.

[11] Shrestha S, Hsu SD, Huang WY, Huang HY, Chen W, Weng SL, Huang HD (2014). A systematic review of microRNA expression profiling studies in human gastric cancer. Cancer Med.

[12] Zhang Z, Li Z, Li Y, Zang A (2014). MicroRNA and signaling pathways in gastric cancer. Cancer Gene Ther.

[13] Motoyama K, Inoue H, Mimori K, Tanaka F, Kojima K, Uetake H, Sugihara K, Mori M (2010). Clinicopathological and prognostic significance of PDCD4 and microRNA-21 in human gastric cancer. International journal of oncology.

[14] Wang Z, Cai Q, Jiang Z, Liu B, Zhu Z, Li C (2014). Prognostic role of microRNA-21 in gastric cancer: a meta-analysis. Med Sci Monit.

[15] Zhang Z, Li Z, Gao C, Chen P, Chen J, Liu W, Xiao S, Lu H (2008). miR-21 plays a pivotal role in gastric cancer pathogenesis and progression. Lab Invest.

[16] Cui H, Wang L, Gong P, Zhao C, Zhang S, Zhang K, Zhou R, Zhao Z, Fan H (2015). Deregulation between miR-29b/c and DNMT3A is associated with epigenetic silencing of the CDH1 gene, affecting cell migration and invasion in gastric cancer. PLoS One.

[17] Gong J, Li J, Wang Y, Liu C, Jia H, Jiang C, Luo M, Zhao H, Dong L, Song W, Wang F, Wang W, Zhang J (2014). Characterization of microRNA-29 family expression and investigation of their mechanistic roles in gastric cancer. Carcinogenesis.

[18] Wang Y, Liu C, Luo M, Zhang Z, Gong J, Li J, You L, Dong L, Su R, Lin H, Ma Y, Wang F, Chen J (2015). Chemotherapy-Induced miRNA-29c/Catenin-delta Signaling Suppresses Metastasis in Gastric Cancer. Cancer research.

[19] Dearth RK, Cui X, Kim HJ, Hadsell DL, Lee AV (2007). Oncogenic transformation by the signaling adaptor proteins insulin receptor substrate (IRS)-1 and IRS-2. Cell Cycle.

[20] Geng Y, Ju Y, Ren F, Qiu Y, Tomita Y, Tomoeda M, Kishida M, Wang Y, Jin L, Su F, Wei C, Jia B, Li Y (2014). Insulin receptor substrate 1/2 (IRS1/2) regulates Wnt/beta-catenin signaling through blocking autophagic degradation of dishevelled2. J Biol Chem.

[21] Han TS, Hur K, Xu G, Choi B, Okugawa Y, Toiyama Y, Oshima H, Oshima M, Lee HJ, Kim VN, Chang AN, Goel A, Yang HK (2015). MicroRNA-29c mediates initiation of gastric carcinogenesis by directly targeting ITGB1. Gut.

[22] Ling H, Pickard K, Ivan C, Isella C, Ikuo M, Mitter R, Spizzo R, Bullock MD, Braicu C, Pileczki V, Vincent K, Pichler M, Stiegelbauer V (2015). The clinical and biological significance of MIR-224 expression in colorectal cancer metastasis. Gut.

[23] Shi C, Yang Y, Xia Y, Okugawa Y, Yang J, Liang Y, Chen H, Zhang P, Wang F, Han H, Wu W, Gao R, Gasche C, Qin H, Ma Y, Goel A (2015). Novel evidence for an oncogenic role of microRNA-21 in colitis-associated colorectal cancer. Gut.

[24] Gailhouste L, Gomez-Santos L, Hagiwara K, Hatada I, Kitagawa N, Kawaharada K, Thirion M, Kosaka N, Takahashi RU, Shibata T, Miyajima A, Ochiya T (2013). miR-148a plays a pivotal role in the liver by promoting the hepatospecific phenotype and suppressing the invasiveness of transformed cells. Hepatology.

[25] Pan L, Huang S, He R, Rong M, Dang Y, Chen G (2014). Decreased expression and clinical significance of miR-148a in hepatocellular carcinoma tissues. Eur J Med Res.

[26] Fujita Y, Kojima K, Ohhashi R, Hamada N, Nozawa Y, Kitamoto A, Sato A, Kondo S, Kojima T, Deguchi T, Ito M (2010). MiR-148a attenuates paclitaxel resistance of hormone-refractory, drug-resistant prostate cancer PC3 cells by regulating MSK1 expression. The Journal of biological chemistry.

[27] Murata T, Takayama K, Katayama S, Urano T, Horie-Inoue K, Ikeda K, Takahashi S, Kawazu C, Hasegawa A, Ouchi Y, Homma Y, Hayashizaki Y, Inoue S (2010). miR-148a is an androgen-responsive microRNA that promotes LNCaP prostate cell growth by repressing its target CAND1 expression. Prostate Cancer Prostatic Dis.

[28] Takahashi M, Cuatrecasas M, Balaguer F, Hur K, Toiyama Y, Castells A, Boland CR, Goel A (2012). The clinical significance of MiR-148a as a predictive biomarker in patients with advanced colorectal cancer. PLoS One.

[29] Zhang H, Li Y, Huang Q, Ren X, Hu H, Sheng H, Lai M (2011). MiR-148a promotes apoptosis by targeting Bcl-2 in colorectal cancer. Cell death and differentiation.

[30] Xia J, Guo X, Yan J, Deng K (2014). The role of miR-148a in gastric cancer. Journal of cancer research and clinical oncology.

[31] Delpu Y, Lulka H, Sicard F, Saint-Laurent N, Lopez F, Hanoun N, Buscail L, Cordelier P, Torrisani J (2013). The rescue of miR-148a expression in pancreatic cancer: an inappropriate therapeutic tool. PLoS One.

[32] Zhang R, Li M, Zang W, Chen X, Wang Y, Li P, Du Y, Zhao G, Li L (2014). MiR-148a regulates the growth and apoptosis in pancreatic cancer by targeting CCKBR and Bcl-2. Tumour Biol.

[33] Lombard AP, Mooso BA, Libertini SJ, Lim RM, Nakagawa RM, Vidallo KD, Costanzo NC, Ghosh PM, Mudryj M (2015). miR-148a dependent apoptosis of bladder cancer cells is mediated in part by the epigenetic modifier DNMT1. Mol Carcinog.

[34] Zhou X, Zhao F, Wang ZN, Song YX, Chang H, Chiang Y, Xu HM (2012). Altered expression of miR-152 and miR-148a in ovarian cancer is related to cell proliferation. Oncol Rep.

[35] Jiang F, Li Y, Mu J, Hu C, Zhou M, Wang X, Si L, Ning S, Li Z (2015). Glabridin inhibits cancer stem cell-like properties of human breast cancer cells: An epigenetic regulation of miR-148a/SMAd2 signaling. Mol Carcinog.

[36] Tao S, He H, Chen Q, Yue W (2014). GPER mediated estradiol reduces miR-148a to promote HLA-G expression in breast cancer. Biochemical and biophysical research communications.

[37] Chen Y, Song Y, Wang Z, Yue Z, Xu H, Xing C, Liu Z (2010). Altered expression of MiR-148a and MiR-152 in gastrointestinal cancers and its clinical significance. J Gastrointest Surg.

[38] Guo SL, Peng Z, Yang X, Fan KJ, Ye H, Li ZH, Wang Y, Xu XL, Li J, Wang YL, Teng Y (2011). miR-148a promoted cell proliferation by targeting p27 in gastric cancer cells. Int J Biol Sci.

[39] Yan J, Guo X, Xia J, Shan T, Gu C, Liang Z, Zhao W, Jin S (2014). MiR-148a regulates MEG3 in gastric cancer by targeting DNA methyltransferase 1. Med Oncol.

[40] Sharma T, Hamilton R, Mandal CC (2015). miR-214: a potential biomarker and therapeutic for different cancers. Future Oncol.

[41] Liu D, Tao T, Xu B, Chen S, Liu C, Zhang L, Lu K, Huang Y, Jiang L, Zhang X, Huang X, Han C, Chen M (2014). MiR-361-5p acts as a tumor suppressor in prostate cancer by targeting signal transducer and activator of transcription-6(STAT6). Biochemical and biophysical research communications.

[42] Wu X, Xi X, Yan Q, Zhang Z, Cai B, Lu W, Wan X (2013). MicroRNA-361-5p facilitates cervical cancer progression through mediation of epithelial-to-mesenchymal transition. Med Oncol.

[43] Porter HA, Perry A, Kingsley C, Tran NL, Keegan AD (2013). IRS1 is highly expressed in localized breast tumors and regulates the sensitivity of breast cancer cells to chemotherapy, while IRS2 is highly expressed in invasive breast tumors. Cancer Lett.

[44] Su W, Xu M, Chen X, Nie L, Chen N, Gong J, Zhang M, Su Z, Huang L, Zhou Q (2015). MiR200c targets IRS1 and suppresses prostate cancer cell growth. Prostate.

[45] Wang Y, Hu C, Cheng J, Chen B, Ke Q, Lv Z, Wu J, Zhou Y (2014). MicroRNA-145 suppresses hepatocellular carcinoma by targeting IRS1 and its downstream Akt signaling. Biochemical and biophysical research communications.

[46] Zhou Y, Feng X, Liu YL, Ye SC, Wang H, Tan WK, Tian T, Qiu YM, Luo HS (2013). Down-regulation of miR-126 is associated with colorectal cancer cells proliferation, migration and invasion by targeting IRS-1 via the AKT and ERK1/2 signaling pathways. PLoS One.

