# Supplementary material for: MicroRNA-1225-5p inhibits proliferation and metastasis of gastric carcinoma through repressing insulin receptor substrate-1 and activation of β-catenin signaling
Source: Oncotarget. 2015 Dec 14;7(4):4647–63. doi: 10.18632/oncotarget.6615 (PMC4826233; doi:10.18632/oncotarget.6615)
Supplement: Supplementary file 1 [file oncotarget-07-4647-s001.pdf]

SUPPLEMENTARY FIGURES AND TABLES

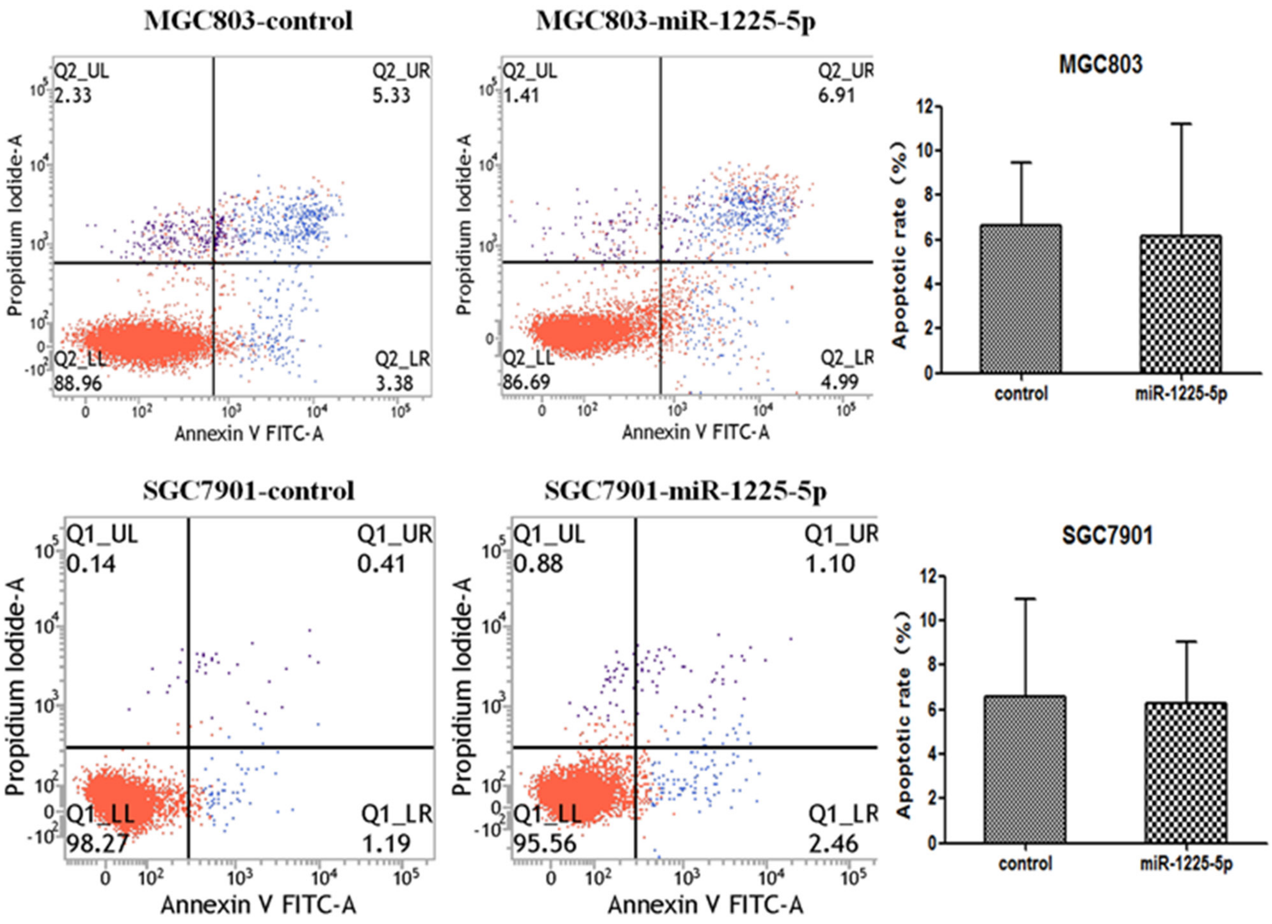

Supplementary Figure S1: miR-1225–5p did no change the cell apoptosis rate of MGC803–1225+ and SGC7901–1225+ cells detected by flow cytometry.

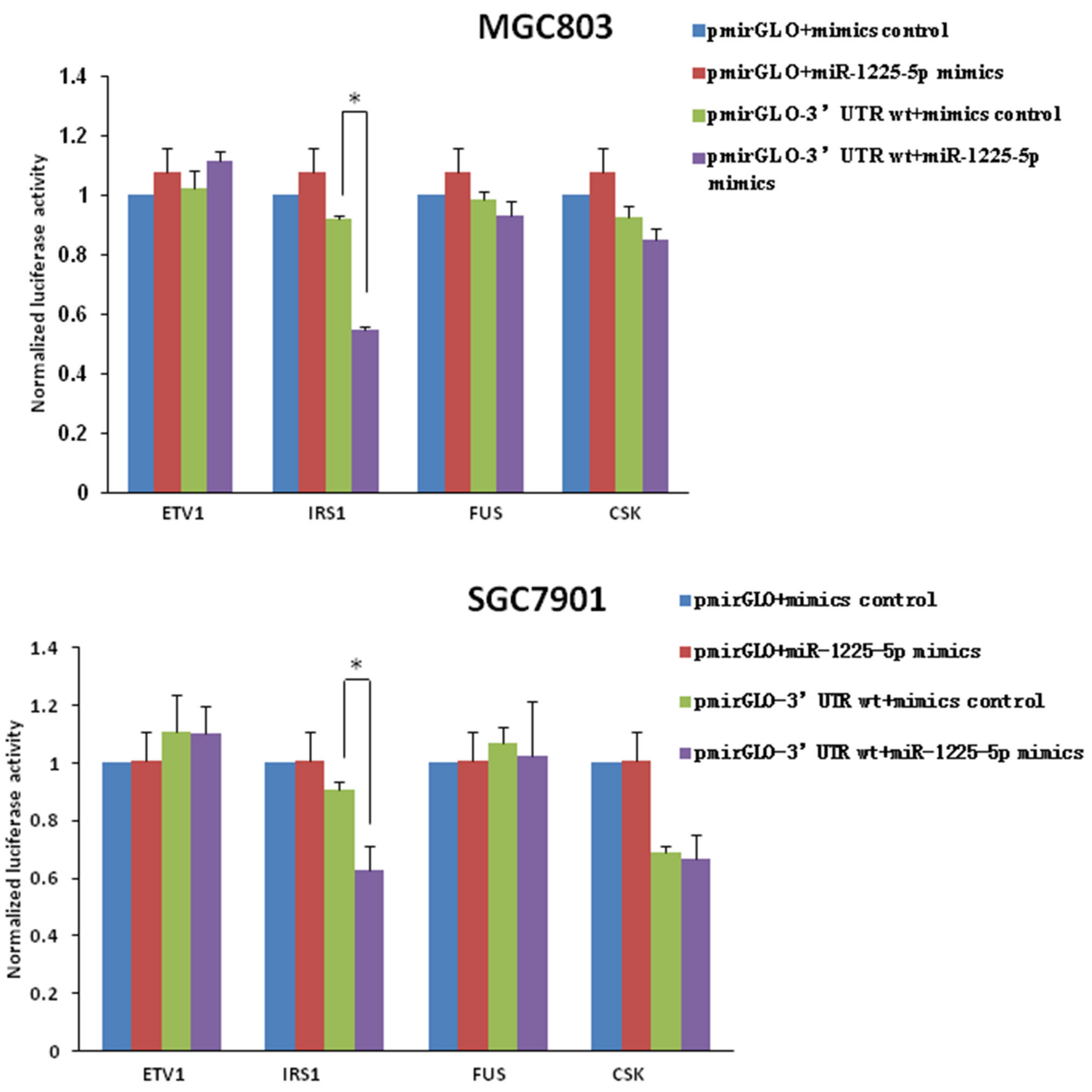

Supplementary Figure S2: Luciferase assay was used to confirm the interaction of miR-1225–5p with its binding site in the 3′-untranslated region (UTR) of the predicted targets, ETV1, IRS1, FUS and CSK.

Supplementary Table S1: Clinical pathological characteristics of 35 patients with gastric carcinomas

Clinical parameters	Number of cases (%)
**Age**	
≤60	12 (34.3)
>60	23 (65.7
**Gender**	
Male	25 (71.4)
Female	10 (28.6)
**Depth of invasion (T)**	
T1	2 (5.7)
T2	8 (22.9)
T3	23 (65.7)
T4	2 (5.7)
**Lymph-node metastasis (N)**	
Negative (N0)	10 (28.6)
Positive (N1~3)	25 (71.4)
**TNM stage**	
I	5 (14.2)
II	10 (28.6)
III	10 (28.6)
IV	10 (28.6)
**Histological type**	
Intestinal	10 (28.6)
Diffuse	25 (71.4)

Supplementary Table S2: Oligonucleotides used for cloning and real-time RT-PCR

Oligonucleotides	Sequences (5′-3′)
3′ UTR **cloning**	
ETV1–3′UTR F	CCGCTCGAGATATCTGTCTCCTTTTCAC
ETV1–3′UTR R	TGCTCTAGAGAAATGCTATCTGTAGTTAACTGCACTGC
FUS-3′UTR F	CCGCTCGAG TTAGCCTGGC TCCCCAGGT
FUS-3′UTR R	TGCTCTAGACCCTTGGGTGATCAGGAATTG
CSK-3′UTR F	CCGCTCGAG CCGCCACTCGCCTTCTTAGA
CSK-3′UTR R	TGCTCTAGAGGACATGGGACACAGAATGGG
IRS1–3′UTR wt F	CCGCTCGAG CTCAACTGGA CATCACAGCA
IRS1–3′UTR wt R	TGCTCTAGACATCGTACCA TCTACTGATG
IRS1–3′UTR mut1 F	TCCTCTTCTAACTCATGGGCATCTAGACTCTAAATATTTCA
IRS1–3′UTR mut1R	CCCATGAGTTAGAAGAGGA
IRS1–3′UTR mut2 F	TCCTCTTCTAACTCATGGGAAGCGAGACTCT AATATTTCA
IRS1–3′UTR mut2R	CCCATGAGTT AGAAGAGGA
**IRS1 cloning**	
IRS1 F	CCGGAATTCGCCACCATGGCGAG CCCTCCGGAG A
IRS1 R	CCCAAGCTT CTGACGGTCC TCTGGCTGC
**Real-time RT-PCR**	
IRS1 F	CTCAACTGGA CATCACAGCA
IRS1 R	CATCGTACCA TCTACTGATG
c-Myc F	TTTCGGGTAGTGGAAAACCA
c-Myc R	CACCGAGTCGTAGTCGAGGT
Cyclin D1 F	TCCTCTCCAAAATGCCAGAG
Cyclin D1 R	GGCGGATTGGAAATGAACTT
GAPDH F	TGCACCACCAACTGCTTAGC
GAPDH R	AGCTCAGGGATGACCTTGCC
